# Effects of a 13-Week Personalized Lifestyle Intervention Based on the Diabetes Subtype for People with Newly Diagnosed Type 2 Diabetes

**DOI:** 10.3390/biomedicines10030643

**Published:** 2022-03-10

**Authors:** Iris M. de Hoogh, Wilrike J. Pasman, André Boorsma, Ben van Ommen, Suzan Wopereis

**Affiliations:** Research Group Microbiology & Systems Biology, TNO, Netherlands Organization for Applied Scientific Research, 3704 HE Zeist, The Netherlands; wilrike.pasman@tno.nl (W.J.P.); andre.boorsma@tno.nl (A.B.); ben.vanommen@tno.nl (B.v.O.); suzan.wopereis@tno.nl (S.W.)

**Keywords:** type 2 diabetes, remission, lifestyle intervention, diet, subtypes, primary care

## Abstract

A type 2 diabetes mellitus (T2DM) subtyping method that determines the T2DM phenotype based on an extended oral glucose tolerance test is proposed. It assigns participants to one of seven subtypes according to their β-cell function and the presence of hepatic and/or muscle insulin resistance. The effectiveness of this subtyping approach and subsequent personalized lifestyle treatment in ameliorating T2DM was assessed in a primary care setting. Sixty participants, newly diagnosed with (pre)diabetes type 2 and not taking diabetes medication, completed the intervention. Retrospectively collected data of 60 people with T2DM from usual care were used as controls. Bodyweight (*p* < 0.01) and HbA1c (*p* < 0.01) were significantly reduced after 13 weeks in the intervention group, but not in the usual care group. The intervention group achieved 75.0% diabetes remission after 13 weeks (fasting glucose ≤ 6.9 mmol/L and HbA1c < 6.5% (48 mmol/mol)); for the usual care group, this was 22.0%. Lasting (two years) remission was especially achieved in subgroups with isolated hepatic insulin resistance. Our study shows that a personalized diagnosis and lifestyle intervention for T2DM in a primary care setting may be more effective in improving T2DM-related parameters than usual care, with long-term effects seen especially in subgroups with hepatic insulin resistance.

## 1. Introduction

The main pathophysiological defects in type 2 diabetes mellitus (T2DM) are insulin resistance (IR) of the liver, muscle, and adipose tissue, and reduced β-cell function (BCF) [1]. Current treatment primarily focuses on lowering blood glucose concentrations and glycated hemoglobin (HbA1c) levels instead of addressing the underlying pathophysiology. Therefore, limited effectiveness may be achieved in diabetes treatment, especially in the longer term [2,3,4]. Several studies have shown that lifestyle interventions have beneficial effects on glycemic control [5,6,7], and may even induce disease remission [8,9]. In the DiRECT trial, a primary-care-led weight management program for T2DM, 46% of the intervention participants achieved disease remission [3,4]. The remission rate appeared related to β-cell capacity [6], indicating that not all persons react similarly to such interventions. As T2DM is a multi-factorial disease affecting multiple organs, and because people differ in their genetics, phenotype, lifestyle, and environment, different mechanisms may underlie T2DM pathophysiology [10,11]. Impaired glucose tolerance (IGT) and impaired fasting glucose (IFG), which are both pre-stages of T2DM, can occur both separately and simultaneously, and differ in prevalence [12]. Moreover, plasma insulin levels in response to an oral glucose tolerance test (OGTT) differ [13]. A greater impairment in first-phase insulin secretion, indicative of hepatic insulin resistance (HIR), can be found in individuals with isolated IFG. People with IGT show higher two-hour insulin and glucose concentrations, indicative of muscle insulin resistance (MIR) [14]. The cardiometabolic T2DM etiologies of systemic low-grade inflammation and lipid dysmetabolism differ between people with MIR and people with HIR [15]. These differences in underlying T2DM pathophysiology may explain the differences in the effectiveness of lifestyle interventions. Indeed, it has been shown that in people with prediabetes with relatively high fasting insulin, a low-fat diet is most effective for weight loss, whereas for people with prediabetes with relatively low fasting insulin, a low-carbohydrate diet is most effective [16]. Another study comparing the two-year effects of both a low-fat and a Mediterranean diet showed a larger improvement in BCF on a low-fat diet in people with HIR, whilst people with MIR or a combination of muscle and liver IR (CIR) benefitted more from a Mediterranean diet [17]. Moreover, it is known that MIR is best counteracted by physical exercise [18], whereas caloric restriction seems to be effective in reducing HIR [19]. Thus, the diabetic subtype can be used to personalize—and potentially increase—the efficacy of and adherence to lifestyle treatment for T2DM.

Herein, we propose a subtyping method that determines an individual’s diabetic phenotype and establishes the underlying pathophysiology [20]. T2DM subtyping was conducted by performing a five-timepoint OGTT, quantifying plasma glucose and insulin concentrations at baseline and 30 min intervals up to two hours. The resulting data were used to determine indices indicative of pancreatic insulin secretion and muscle and liver insulin resistance.

Based on the T2DM subtype, a personalized diagnosis and subsequent tailored treatment were determined. Next, we assessed the effectiveness of this T2DM subtyping approach in ameliorating T2DM by the evaluation of HbA1c and fasting plasma glucose (FPG), as well as the associated risk factors, including body weight, in comparison to usual care. Additionally, we elucidated whether personalized interventions improved the diabetic phenotype and induced diabetes remission. This study took place in a primary care setting to assess the feasibility of this more personalized approach in a real-life setting. The intervention lasted 13 weeks, with a two-year follow-up.

## 2. Materials and Methods

### 2.1. Study Population

Eighty-two participants with prediabetes or newly diagnosed T2DM (within the last 12 months), according to the Dutch general practitioners’ standards, were recruited from eight primary care centers in Hillegom, The Netherlands. In The Netherlands, T2DM diagnosis is determined based on glucose values with two FPG of ≥7.0 mmol/L or one FPG of ≥7.0 mmol/L combined with non-FPG of ≥11.1 mmol/L on two different days, whereas prediabetes is defined as an FPG of ≥6.1 and <7.0 mmol/L and/or a non-FPG of ≥7.8 and <11.1 mmol/L. Participants were eligible for study participation if they were aged 30–80 years, and had a stable body mass index (BMI) between 25 and 35 kg/m^2^. The exclusion criteria were the use of plasma glucose-lowering medication within the past year, the use of systemic corticosteroids and β-blockers in the past month, pancreatic or (late-onset) type 1 diabetes, and other medical conditions, including gastrointestinal dysfunction, psychiatric disorders, severe hypertension, and renal insufficiency. Of the 82 participants initially enrolled in the study, 16 were excluded after the baseline OGTT because of either very poor BCF (*n* = 8) or normal glucose metabolism (neither reduced BCF nor IR) (*n* = 8). 

From the same primary care center, the data of 60 people with prediabetes or newly diagnosed T2DM in usual care, meeting the above-stated inclusion and exclusion criteria, i.e., aged 30–80 years, BMI between 25 and 35 kg/m^2^, and no use of plasma glucose-lowering medication, were collected retrospectively as controls. The historic data included fewer people with prediabetes because there is no official monitoring protocol for prediabetes according to the Dutch general practitioners’ standards [21], as a result of which registration and monitoring occurs less frequently.

All participants gave written informed consent. The study protocol was approved by the Medical Ethics Committee Brabant (NL48742.028.14). The study was performed in accordance with the Declaration of Helsinki and good clinical practice and was registered at ClinicalTrails.gov (NCT02196350).

### 2.2. Study Design

**Figure 1:**
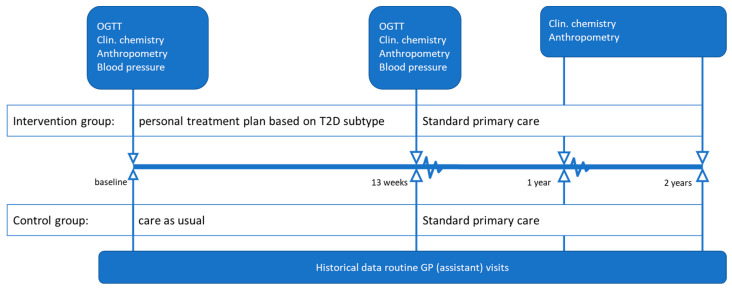
Study design. GP = general practitioner; OGTT = oral glucose tolerance test; clin. chemistry = clinical chemistry (HbA1c, triglycerides and HDL, LDL, and total cholesterol). Anthropometry includes body height (only at baseline), body weight, waist circumference, and fat percentage.

This study was exploratory. At baseline, clinical chemistry, blood pressure, and anthropometric measurements (length, body weight, waist circumference, and fat percentage) were performed. Based on the T2DM subtype, participants were allocated to one of seven personalized lifestyle treatments. The 13-week intervention was supervised by a dietician and/or physiotherapist. All participants visited the general practitioner’s assistant at baseline and in weeks 4, 8, and 13, and participants visited the dietician at baseline and in weeks 1, 2, 6, 10, and 13 for (personalized) dietary advice. Those participants allocated to a treatment including exercise visited the physiotherapist for supervised personalized exercise training three times a week for 13 weeks. After the 13-week intervention, the measurements were repeated, including an OGTT to determine changes in glucose metabolism and the T2DM subtype. After the 13-week intervention, the participants returned to standard primary care. Anthropometry and clinical chemistry were repeated one and two years after baseline. Healthcare providers were instructed to be reluctant in prescribing oral diabetes medication or insulin therapy during the study. The intervention group was compared with historic data from a control group that received usual care according to the Dutch general practitioners’ standards [21]. This states to start with prescribing oral diabetes medication when the HbA1c target level of 7.0% (53 mmol/mol) is not reached with a non-drug treatment. For this study, diabetes remission was defined as: (a) Fasting glucose ≤ 6.9 mmol/L, (b) HbA1c < 6.5% (48 mmol/mol), (c) no use of glucose-lowering medication, and (d) meeting these targets at the 12- and 24-month follow-up [2]. Figure 1 provides an overview of the study design. 

### 2.3. Clinical Chemistry and OGTT

After an overnight fast, blood samples were taken before (0 min) and at four time points after drinking a 75 g glucose solution (t = 30, 60, 90, and 120 min) to determine plasma glucose and insulin concentrations. HbA1c and lipids were assessed at baseline, 13 weeks, and at the one- and two-year follow-up. OGTT and blood sampling were performed at the service center Elsbroek by AtalMedial in Hillegom, The Netherlands. Lab analyses were performed by AtalMedials’ lab located in Spaarne Gasthuis Hospital, The Netherlands.

### 2.4. Subtyping Rationale

Glucose and insulin response to the OGTT was used to calculate the following indices: Disposition Index (DI) [22,23,24], Matsuda Index, Hepatic Insulin Resistance Index (HIRI) [25], and Muscle Insulin Sensitivity Index (MISI) [26]. Cut-off values for these indices, to distinguish between healthy and diabetic scores, were determined using data from ~1100 participants [27,28,29]. These cut-offs were calculated and validated using different subsets of healthy participants, participants with prediabetes (IFG, IGT, or both), and people with undiagnosed and clinically diagnosed T2DM.

After calculating the indices, participants were assigned to one of seven subtypes according to BCF (moderate or low) and the presence of hepatic IR and/or muscle IR (Supplemental Table S1). Individuals with no IR and no BCF were excluded at baseline. If, after the intervention, participants reverted to no IR and no BCF, these participants were assigned to the “healthy” subtype.

### 2.5. Interventions

The HIR and CIR subgroup received a very-low-calorie diet (VLCD) for one week, using meal replacements (Modifast) three times a day (500 kcal/day), followed by a 12-week low-calorie diet (LCD; 1000 kcal/day) based on a personal meal plan provided by a dietician. Participants could opt for meal replacements for a maximum of one meal per day. Groups with poor BCF (PB), PB-HIR, or PB-CIR received 13 weeks of LCD, similar to the LCD of the HIR and CIR subgroups. Groups with MIR or PB-MIR followed an isocaloric diet (ICD), comprising normal food products. 

In addition to the dietary intervention, participants in the HIR, PB, and PB-HIR subgroup were stimulated to adhere to the Dutch Norm for Healthy Physical Activity for overweight people (moderate exercise of 60 min/day). The CIR and PB-CIR subgroup were stimulated to adhere to the Dutch Norm for Physical Activity for one week, followed by 12 weeks of strength and endurance training (thrice a week for 60 min), supervised by a physiotherapist. The MIR and PB-MIR subgroups performed supervised strength and endurance training for 13 weeks.

### 2.6. Statistical Analysis

Complete case analysis was performed using only paired data at baseline and after 13 weeks. Two weighted linear mixed models were created, from which all statistical results were subsequently derived, using the “lmer” package [30]. One model included subtype as the main effect, whereas the other contained group. Both models included time as the main effect and the interaction of time with either group and subtype. Furthermore, both models included the participant as a random factor. When fitting the models, statistical outliers were excluded when their standardized model residuals were further than three standard deviations away from 0. When applying these models, some variables were log10-transformed to account for heteroscedasticity in the model residuals.

Type-III sum-of-squares *p*-values were calculated for the main effects using the “car” package, whereas *p*-values for the post hoc tests were calculated using the “emmeans” package [31]. Additionally, *p*-values of <0.05 were deemed statistically significant. The R Project for Statistical Computing software version 3.4.3 for Windows (The R Project for Statistical Computing, Auckland City, Auckland, New Zealand) was used for statistical analysis [32].

## 3. Results

### 3.1. Baseline Characteristics

A total of 60 out of the 66 participants completed the intervention. At baseline, the intervention group had significantly lower HbA1c and FPG and significantly higher BMI compared with the usual care group (Table 1). Moreover, age tended to be higher in the usual care group *(p =* 0.06). In both groups, the average HbA1c levels were below the target level for people with type 2 diabetes, which is 7% (53 mmol/mol) in The Netherlands [21].

### 3.2. Intervention Effects Compared with Usual Care

After 13 weeks, body weight (*p* < 0.001) and HbA1c (*p* < 0.001) were significantly lower compared with baseline in the intervention group, whilst there were no significant changes in the usual care group (Supplemental Table S2). After one and two years of follow-up, body weight (*p* < 0.001) and HbA1c (*p* < 0.001 at one year and *p* < 0.01 at two years) remained significantly lower compared with baseline in the intervention group. In the usual care group, body weight (*p* < 0.01) was reduced compared with baseline at the two-year follow-up only. 

In the intervention group, total cholesterol (−0.47 mmol/L; *p* < 0.01), triglycerides (−0.58 mmol/L; *p* < 0.001), and waist circumference (−11 cm; *p* < 0.001) decreased after the intervention. These data were not available for the usual care group, as these markers are not measured regularly in usual care. 

### 3.3. Diabetes Remission

Table 2 shows the fraction of participants who were classified as “in remission” for the usual care and intervention group. The results are shown as the fraction of participants diagnosed with T2DM at baseline, as our study population also included participants with prediabetes. The intervention group achieved significantly more T2DM remission after 13 weeks compared with the usual care group (*p* = 0.0002). In the intervention group, two participants started using glucose-lowering medication during the follow-up period of the study. For the usual care group, no medication data were available for follow-up, so it was unclear what proportion of participants were still in remission at the one- and two-year follow-ups. 

For the intervention group, participants that achieved remission after 13 weeks showed significantly more weight loss than participants that did not achieve remission (−10.7 kg resp. −4.6 kg; *p* < 0.001).

Of the participants with prediabetes at baseline, 89% remained prediabetic, whilst 11% progressed to T2DM during the study. Those participants that progressed to T2DM showed significantly less weight loss than participants that remained prediabetic (*p* = 0.05).

### 3.4. Changes in the Diabetic Phenotype in the Intervention Group

**Figure 2:**
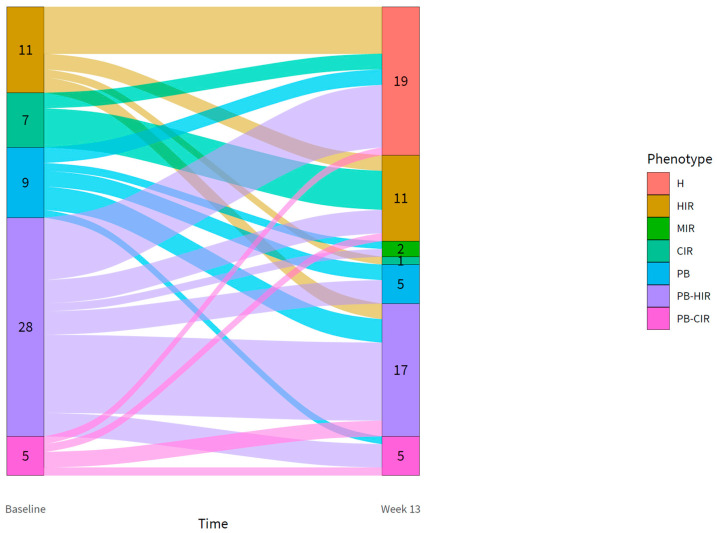
Flow diagram showing the shift in subtypes for participants from baseline to 13 weeks. A shift upwards illustrates a shift toward a less complex phenotype. H = healthy; HIR = moderate BCF and liver IR; MIR = moderate BCF and muscle IR; CIR = moderate BCF and combined IR; PB = low BCF and no IR; PB-HIR = low BCF and liver IR; PB-CIR = low BCF and combined IR.

At baseline, 11 participants had hepatic IR (HIR), 7 participants had muscle and hepatic IR (combined IR; CIR), 9 participants had isolated poor BCF (PB), 28 participants had PB-HIR, and 5 participants had PB-CIR. At baseline, there were no participants with a healthy, MIR, or PB-MIR subtype. A substantial redistribution of participants over the subtypes was found after 13 weeks of intervention (Figure 2). The most noticeable trend was seen for the HIR subtype, with 55% of the participants converting into a healthy subtype after the intervention. For the PB-HIR and CIR subtype this was 29%, whereas for the PB and PB-CIR subtypes, it was 22% and 20%, respectively.

In total, 32% of the participants (*n* = 19) obtained a healthy subtype (normal BCF without IR) after 13 weeks of intervention, of which 7 participants met the criteria for T2DM remission, 11 had prediabetes at baseline, and one participant reached a HbA1c of 5.4% (36 mmol/mol) and FPG of 7.0 mmol/L at 13 weeks. Including the participant with borderline remission and a healthy subtype, in total, 10 participants could be classified as T2DM after 13 weeks of intervention, of which 6 had the PB-HIR subtype and 3 the PB-CIR subtype.

### 3.5. Changes in the Glucose Metabolism in the Intervention Group

Liver IR significantly improved (HIRI of −80.2; *p* < 0.001) after 13 weeks. Postprandial glucose (PPG) decreased significantly (−1.34 mmol/L; *p* < 0.001) after 13 weeks. The disposition index and MISI did not change over time (*p* = 0.231, resp. *p* = 0.945). 

Furthermore, the 13-week intervention significantly decreased HIRI in all subtypes with liver IR (unknown for subgroup PB-CIR, as no *p*-value could be calculated due to missing data; Table 3). FPG decreased in two of these subgroups (HIR and PB-HIR). 

Unexpectedly, MISI increased in the PB-HIR subgroup, indicating a decrease in muscle insulin sensitivity, although the mean MISI was still within the healthy range (−2.87 ± 1.27). Postprandial glucose improved in the CIR subgroup only. The disposition index only improved in the PB-HIR subgroup. 

### 3.6. Long-Term Intervention Effects

All subgroups showed a significant reduction in body weight after the intervention, which was maintained at one and two years of follow-up for all subgroups, except for the group with PB-CIR (+3.3 kg; *p* < 0.05) (Table 4).

In the HIR and PB-HIR subgroups, FPG and HbA1c decreased after the intervention, which was maintained up to two years of follow-up. In the CIR subgroup, HbA1c was significantly reduced after the intervention, but this effect was not maintained at follow-up. For all other subgroups, no significant changes in FPG or HbA1c were found.

## 4. Discussion

In this study, we showed that diabetes subtyping and subsequent tailored lifestyle interventions in a primary care setting are more effective in improving T2DM-related parameters than usual care. Bodyweight and HbA1c were significantly reduced after 13 weeks of intervention, whilst no changes in these markers were seen with usual care. Additionally, the improvements in health status were maintained up to two years after the intervention. Our results suggest that a (V)LCD may be more effective in improving liver IR, whilst resistance training may be more effective in improving muscle IR. 

Unique to our study was the use of an extended OGTT in a primary care setting for identifying the diabetic phenotype and subsequently using this knowledge for a tailored lifestyle treatment. Various clinical studies have shown that persons with T2DM may differ in their metabolic profile, resulting in differential responses to lifestyle interventions [13,15,17,33,34,35,36]. However, in these studies, participants were assigned to a dietary pattern at random and subtype effects were identified retrospectively. To the best of our knowledge, this is the first study in which the metabolic profiling of people with T2DM was performed prospectively and used for a phenotype-based sub-diagnosis and adjacent tailored lifestyle treatment in a real-life primary care setting. 

Our results suggest that these tailored treatments indeed induce differential effects. The specific improvements in HIRI and FPG for the groups with liver IR, and the improvements in PPG in the groups with combined IR (CIR), suggest that the tailored treatment may have added value over a one-size-fits-all approach. However, as there were no participants with isolated muscle IR (with or without low BCF), future research is needed to investigate the effects of a solely physical activity intervention in people with muscle IR. Previous research has shown that improving MISI is more difficult or may take longer [36,37]. The lack of effect, or even a small negative effect in the PB-HIRI group, on MISI in our study may be a result of weight loss. Weight loss may have included a loss of muscle mass, which may negatively affect MISI. However, in the PROBE trial, the lack of effect on MISI coincided with an improved muscle mass [36]. 

Average weight loss in our study after one year was 7.1 kg, and this resulted in improvements in T2DM-related health parameters. Caloric restriction has been shown to reduce pancreatic and hepatic fat content and hepatic IR and improve BCF [19,38]. In our study, weight loss was strongly correlated with achieving remission, with an average weight loss of −10.7 kg in the group that achieved remission and −4.7 kg in the group without remission. These results indicate a relationship between T2DM remission achievement and weight loss, as also shown in the DiRECT trial [3]. Modest weight loss of 5–10% has also been previously linked to improvements in cardiovascular risk factors, including HbA1c [39]. Non-responders predominantly had a complex phenotype with combined IR, decreased BCF, or both, indicating that achieving remission is more difficult with a more progressed disease status. Karter et al. observed an association between the rate of remission and years since diagnosis [40], and Taylor et al. linked non-response to a lifestyle intervention to a more advanced, irreversible stage of β-cell dysfunction. In our study, subgroups with combined insulin resistance with or without poor BCF or poor BCF only (CIR, PB, and PB-CIR) showed only short-term or no improvements in FPG or HbA1c [6], whereas subgroups with hepatic insulin resistance with or without decreased BCF (HIR and PB-HIR) had long-term improvements in FPG and HbA1c. Interestingly, persons with combined insulin resistance (CIR) did achieve a sustained bodyweight reduction of 7 kg after two years of follow-up that did not result in reduced hyperglycemia. Therefore, the subgroups with isolated liver IR (HIR and PB-HIR) benefitted most from the lifestyle treatment, as shown by improved bodyweight, HIRI, FPG, and HbA1c after the intervention period, and improved FPG and HbA1c after one and two years of follow-up. 

The percentage of participants in remission after the intervention was 75.0%. However, when looking at the diabetic phenotype of the included participants, based on indices for organ-specific insulin sensitivity and β-cell function, only 28% of the participants with T2DM at baseline had a fully remitted and healthy subtype (normal BCF and no IR) after the intervention. T2DM is indeed a multi-factorial disease affecting multiple organs, and normalization of HbA1c and/or FPG levels can still coincide with reduced organ function and β-cell dysfunction [41]. It is therefore recommended for individuals who achieve remission to remain under the supervision of healthcare professionals [42].

Increasing focus on the functioning of organs involved in the pathophysiology of T2DM (liver, adipose tissue, skeletal muscle, and pancreas) may therefore provide more insight into the effects of interventions and disease status, instead of merely focusing on remission numbers. We therefore suggest performing an extended OGTT to assess diabetes pathophysiology so that disease progression or regression before and after an intervention can be more accurately determined over time. Indeed, in a pilot study using the same subtyping methodology in a population with a longer T2DM disease duration, none of the participants were able to achieve a healthy subtype, even though improvements in HbA1c and FPG were observed [37]. Additionally, the diabetes subtyping methodology allows for a more tailored lifestyle intervention, which may improve intervention success. For this, our subtyping method can be used, which uses blood glucose and insulin response to a five-point OGTT as a measure of diabetes pathophysiology [20,43]. Besides our subtyping model, other models exist, using established T2DM genetic loci to identify several diabetic phenotypes [44], using clinical parameters to cluster adult-onset diabetes [45,46], or using patterns of specific glycemic responses called “glucotypes” [47]. The importance of differences in organ function was also suggested in the Diogenes and Maastricht studies, which showed an altered metabolic profile in persons with obesity and liver IR compared with persons with obesity and muscle IR [15,48,49]. However, our subtyping method is, to the best of our knowledge, the first that provides a complete picture of the underlying pathophysiology of T2DM and offers the opportunity for tailored treatment. 

A few limitations need to be discussed. An important limitation of this explorative study was that participants in the usual care group were not accurately matched with the intervention group for BMI and age, due to a limited available patient database. Additionally, or maybe consequently, the usual care group had higher baseline FPG and HbA1c values compared with the intervention group. Additionally, as OGTTs are not performed in usual care, no data on type 2 diabetes subtypes and the comparability of the distribution thereof with the intervention group were available. Considering the higher baseline FPG and HbA1c values, the participants in the usual care group, although newly diagnosed, without treatment for type 2 diabetes and with an average HbA1c level below the target, could all have had a poor BCF, which would explain the scarce response in this group. Furthermore, no data on medication were available for the usual care group, except for baseline, where oral medication was used as the exclusion criterium. Possibly, in the usual care group, the use of glucose-lowering medication could have started throughout the trial. These differences between the usual care and intervention groups may have influenced our results. A future efficacy study with a prospective control arm randomized for BMI, age, FPG, HbA1c, and BCF status, as well as careful registration of medicine use is needed to confirm the current results from diabetes subtyping and tailored lifestyle intervention.

In The Netherlands, people are screened for T2DM by determining FPG levels and sometimes HbA1c levels. As 2 h blood glucose is not measured, participants with isolated IGT, which is defined as 2 h glucose levels of 7.8–11.1 mmol [50], are missed. This may have caused the underrepresentation of participants with muscle IR in our study. To improve the early detection of and treatment for participants with isolated IGT, we suggest always performing an OGTT or at least measuring 2 h blood glucose levels. 

In the intervention group, the number of participants was relatively small per diabetes subtype, especially for the PB-CIR, CIR, and PB groups. Despite the small diabetes subtype groups, we were still able to reach statistical significance for some of the variables, providing interesting insights into the underlying pathophysiology of type 2 diabetes and how lifestyle interventions can interact with this. For a follow-up study, a larger study population is required to confirm and validate these findings. It will remain difficult, however, to influence equal distribution over the diabetes subtypes, as this follows from the OGTT. Lastly, the frequency of visits to a healthcare professional, including visits to the GP assistant, as well as to dieticians and/or physiotherapists, was probably lower in the control group as compared with the intervention group, which could have resulted in differences in intervention adherence, thereby affecting the study results. However, this more intensive guidance, as well as referral to lifestyle professionals such as dieticians and physiotherapists may be required to help people with newly diagnosed T2DM to initiate behavior change.

## 5. Conclusions

This was the first study to provide tailored treatment based on the diabetic phenotype of people with T2DM in a primary care setting. The tailored approach resulted in differential effects on T2DM phenotypes, with the largest and most persistent improvement in participants with isolated liver IR (with or without low BCF). Our results suggest that a (V)LCD may be more effective in improving liver IR, whilst resistance training may be more effective in improving muscle IR. Future research, including participants with isolated muscle IR, a prospective control arm matching the intervention arm, and a larger number of study participants to have larger subgroups of diabetes subtypes, should confirm these findings. Even though diabetes remission was achieved by the majority of participants in the intervention group, organ-specific IR and BCF were not fully recovered. This calls for continued monitoring to avoid relapse, and long-term adherence to a tailored-lifestyle treatment may be required for people who achieve T2DM remission. Lastly, this study showed that the tailored approach can be implemented in current primary care and can result in remission or reversal of the disease in the first three months after T2DM diagnosis.

## Figures and Tables

**Table 1:** Baseline characteristics by treatment group.

Characteristic	Usual Care	Intervention	*p*-Value
*n*	60	60	
Men/women (*n*)	34/26	29/31	NS
Age (years)	65.2 ± 9.7	63.4 ± 7.9	0.06
Body height (m)	1.73 ± 0.10	1.72 ± 0.10	NS
Bodyweight (kg)	90.4 ± 15.1	96.3 ± 16.1	NS
BMI	29.9 ± 5.0	32.6 ± 4.8	0.035
HbA1c (%) HbA1c (mmol/mol)	6.7 ± 3.4	6.0 ± 2.8	<0.001
	49.7 ± 13.9	42.6 ± 7.4	
FPG (mmol/L)	8.3 ± 4.0	7.0 ± 1.5	0.005
SBP (mmHg)	136 ± 19	137 ± 14	NS
DBP (mmHg)	82 ± 11	83 ± 10	NS
Total cholesterol (mmol/L)	5.9 ± 1.9 ^†^	5.7 ± 1.1	NS
HDL-cholesterol (mmol/L)	1.3 ± 0.5 ^†^	1.3 ± 0.3	NS
Triglycerides (mmol/L)	3.5 ± 5.4 ^†^	2.2 ± 1.0	NS

Data are the mean ± standard deviation, unless otherwise indicated. ^†^ *n* ≈ 20, not available for all controls and after outlier removal. BMI = body mass index; HbA1c = glycated hemoglobin; FPG = fasting plasma glucose; SBP = systolic blood pressure; DBP = diastolic blood pressure; HDL = high-density lipoprotein; NS = not significant.

**Table 2:** Remission data * for the intervention and usual care groups after the intervention (week 13) and at the one- and two-year follow-ups (week 52 and 104), expressed as the number and percentage of participants with T2DM at baseline **.

	Usual Care		Intervention	
	(*n* = 41)	(%)	(*n* = 25)	(%)
13 weeks	5	22.0	19	75.0
52 weeks	-	-	13	52.4
104 weeks	-	-	7	28.6

* Remission was defined as fasting plasma glucose ≤ 6.9 mmol/L and HbA1c < 6.5% (48 mmol/mol), no use of glucose-lowering medication, and meeting these targets at 12 and 24 months of follow-up; medication data were not available at follow-up for the usual care group. ** In other words, subjects with prediabetes at baseline were excluded from this table, as the remission definition does not apply to people with prediabetes.

**Table 3:** Changes in oral glucose tolerance test response from baseline to 13 weeks (end of intervention) for the main type 2 diabetes subtypes.

Subtype	FPG	PPG	DI	HIRI	MISI
HIR (*n* = 11)	−1.2 **	−1.1	2.19	−1145 **	0.41
CIR (*n* = 7)	−0.3	−3.1 *	1.44	−619 *	−1.71 ^†^
PB (*n* = 9)	0.3	0.2	0.33	138	0.09
PB-HIR (*n* = 28)	−1.2 **	−0.3	0.80 *	−22 **	1.58 **
PB-CIR (*n* = 5)	−0.6	−8.4 ^†^	0.87	2525 ^†^	−2.16 ^‡^

The data are deltas between baseline and 13 weeks of intervention. FPG = fasting plasma glucose; PPG = postprandial glucose; DI = disposition index; HIRI = hepatic insulin resistance index; MISI = muscle insulin sensitivity index; HIR = moderate BCF and liver IR; CIR = moderate BCF and combined IR; PB = low BCF and no IR; PB-HIR = low BCF and liver IR; PB-CIR = low BCF and combined IR. * *p* < 0.01 and ** *p* < 0.001 compared with baseline; ^†^ no *p*-value available due to missing data; ^‡^ trend toward a decrease (*p* = 0.0590).

**Table 4:** Changes in body weight, FPG, and HbA1c from baseline (week 0) to the end of the intervention (week 13) and to the one- and two-year follow-ups (weeks 52 and 104) for the type 2 diabetes subtypes.

	HIR (*n* = 11)	CIR (*n* = 7)	PB (*n* = 9)	PB-HIR (*n* = 28)	PB-CIR (*n* = 5)
Bodyweight (kg)					
Weeks 0–13	−10.2 ***	−13.1 ***	−5.6 **	−8.8 ***	−5.7 *
Weeks 0–52	−9.1 ***	−7.3 **	−4.8 ***	−6.0 ***	2.0
Weeks 0–104	−8.4 ***	−7.1 **	−2.3 *	−6.0 ***	3.3 *
Fasting glucose (mmol/L)					
Weeks 0–13	−1.1 ***	−0.3	0.3	−1.1 ***	−0.5
Weeks 0–52	−1.3 ***	−0.2	0.0	−0.7 ***	0.4
Weeks 0–104	−1.0 ***	−0.2	0.4	−0.7 ***	−0.3
HbA1c (mmol/mol)					
Weeks 0–13	−3.4 ***	−3.3 *	0.0	−6.2 ***	−2.2
Weeks 0–52	−4.3 **	−1.3	−1.3	−4.9 ***	−1.5
Weeks 0–104	−2.4 *	−0.4	1.8	−2.5 **	−1.3

The data are deltas comparing baseline to week 13 (end of intervention), week 52 (one year follow-up), and week 104 (two years follow-up). HIR = moderate BCF and liver IR; CIR = moderate BCF and combined IR; PB = low BCF and no IR; PB-HIR = low BCF and liver IR; PB-CIR = low BCF and combined IR. * *p* < 0.05, ** *p* < 0.01, and *** *p* < 0.001 compared with baseline.

## Data Availability

The data presented in this study are available on reasonable request from the corresponding author.

## Supplementary Materials

The following are available online at https://www.mdpi.com/article/10.3390/biomedicines10030643/s1: Table S1: Deviating indices and associated treatment plan for all seven type 2 diabetes subtypes and the “healthy” subgroup; Table S2: Means (SDs) and significant changes in the variables after 13 weeks of intervention and one or two years of follow-up for the usual care and intervention groups.

## References

[1] Defronzo R.A. (2009). From the triumvirate to the ominous octet: A new paradigm for the treatment of type 2 diabetes mellitus. Diabetes.

[2] Buse J.B., Caprio S., Cefalu W.T., Ceriello A., Del Prato S., Inzucchi S.E., McLaughlin S., Phillips G.L., Robertson R.P., Rubino F. (2009). How Do We Define Cure of Diabetes?. Diabetes Care.

[3] Lean M.E., Leslie W.S., Barnes A.C., Brosnahan N., Thom G., McCombie L., Peters C., Zhyzhneuskaya S., Al-Mrabeh A., Hollingsworth K.G. (2018). Primary care-led weight management for remission of type 2 diabetes (DiRECT): An open-label, cluster-randomised trial. Lancet.

[4] Lean M.E.J., Leslie W.S., Barnes A.C., Brosnahan N., Thom G., McCombie L., Peters C., Zhyzhneuskaya S., Al-Mrabeh A., Hollingsworth K.G. (2019). Durability of a primary care-led weight-management intervention for remission of type 2 diabetes: 2-year results of the DiRECT open-label, cluster-randomised trial. Lancet Diabetes Endocrinol..

[5] Colberg S.R. (2012). Physical activity: The forgotten tool for type 2 diabetes management. Front. Endocrinol..

[6] Taylor R., Al-Mrabeh A., Zhyzhneuskaya S., Peters C., Barnes A.C., Aribisala B.S., Hollingsworth K.G., Mathers J.C., Sattar N., Lean M.E.J. (2018). Erratum: Remission of Human Type 2 Diabetes Requires Decrease in Liver and Pancreas Fat Content but Is Dependent upon Capacity for β Cell Recovery. Cell Metab..

[7] Knowler W.C., Barrett-Connor E., Fowler S.E., Hamman R.F., Lachin J.M., Walker E.A., Nathan D.M., Diabetes Prevention Program Research Group (2002). Reduction in the incidence of type 2 diabetes with lifestyle intervention or metformin. N. Engl. J. Med..

[8] Steven S., Hollingsworth K.G., Al-Mrabeh A., Avery L., Aribisala B., Caslake M., Taylor R. (2016). Very Low-Calorie Diet and 6 Months of Weight Stability in Type 2 Diabetes: Pathophysiological Changes in Responders and Nonresponders. Diabetes Care.

[9] Ried-Larsen M., Johansen M.Y., MacDonald C.S., Hansen K.B., Christensen R., Wedell-Neergaard A., Pilmark N.S., Langberg H., Vaag A.A., Pedersen B.K. (2019). Type 2 diabetes remission 1 year after an intensive lifestyle intervention: A secondary analysis of a randomized clinical trial. Diabetes Obes. Metab..

[10] Kahn S.E., Cooper M.E., Del Prato S. (2014). Pathophysiology and treatment of type 2 diabetes: Perspectives on the past, present, and future. Lancet.

[11] Galicia-Garcia U., Benito-Vicente A., Jebari S., Larrea-Sebal A., Siddiqi H., Uribe K.B., Ostolaza H., Martín C. (2020). Pathophysiology of Type 2 Diabetes Mellitus. Int. J. Mol. Sci..

[12] Meigs J.B., Muller D.C., Nathan D.M., Blake D.R., Andres R., Baltimore Longitudinal Study of Aging (2003). The natural history of progression from normal glucose tolerance to type 2 diabetes in the Baltimore Longitudinal Study of Aging. Diabetes.

[13] Pearson E.R. (2019). Type 2 diabetes: A multifaceted disease. Diabetologia.

[14] Abdul-Ghani M.A., Tripathy D., DeFronzo R.A. (2006). Contributions of β-cell dysfunction and insulin resistance to the pathogenesis of impaired glucose tolerance and impaired fasting glucose. Diabetes Care.

[15] Van Der Kolk B.W., Kalafati M., Adriaens M., Van Greevenbroek M.M.J., Vogelzangs N., Saris W.H.M., Astrup A., Valsesia A., Langin D., Van Der Kallen C.J.H. (2019). Subcutaneous adipose tissue and systemic inflammation are associated with peripheral but not hepatic insulin resistance in humans. Diabetes.

[16] Hjorth M.F., Astrup A., Zohar Y., Urban L.E., Sayer R.D., Patterson B.W., Herring S.J., Klein S., Zemel B.S., Foster G.D. (2019). Personalized nutrition: Pretreatment glucose metabolism determines individual long-term weight loss responsiveness in individuals with obesity on low-carbohydrate versus low-fat diet. Int. J. Obes..

[17] Blanco-Rojo R., Alcala-Diaz J.F., Wopereis S., Perez-Martinez P., Quintana-Navarro G.M., Marin C., Ordovas J.M., van Ommen B., Perez-Jimenez F., Delgado-Lista J. (2016). The insulin resistance phenotype (muscle or liver) interacts with the type of diet to determine changes in disposition index after 2 years of intervention: The CORDIOPREV-DIAB randomised clinical trial. Diabetologia.

[18] Kirwan J.P., Solomon T.P.J., Wojta D.M., Staten M.A., Holloszy J.O. (2009). Effects of 7 days of exercise training on insulin sensitivity and responsiveness in type 2 diabetes mellitus. Am. J. Physiol. Metab..

[19] Lim E.L., Hollingsworth K.G., Aribisala B.S., Chen M.J., Mathers J.C., Taylor R. (2011). Reversal of type 2 diabetes: Normalisation of beta cell function in association with decreased pancreas and liver triacylglycerol. Diabetologia.

[20] Van Ommen B., Wopereis S., van Empelen P., van Keulen H.M., Otten W., Kasteleyn M., Molema J.J.W., de Hoogh I.M., Chavannes N.H., Numans M.E. (2018). From diabetes care to diabetes cure-the integration of systems biology, ehealth, and behavioral change. Front. Endocrinol..

[21] Barents E., Bilo H., Donk M., Hart H., Verburg-Oorthuizen A., Wiersma T. (2018). NHG-Standaard Diabetes Mellitus Type 2-Pagina 1 NHG-Standaard Diabetes Mellitus Type 2 (M01). https://richtlijnen.nhg.org/standaarden/diabetes-mellitus-type-2.

[22] Bergman R.N., Phillips L.S., Cobelli C. (1981). Physiologic evaluation of factors controlling glucose tolerance in man: Measurement of insulin sensitivity and beta-cell glucose sensitivity from the response to intravenous glucose. J. Clin. Investig..

[23] Kahn S.E., Prigeon R.L., McCulloch D.K., Boyko E.J., Bergman R.N., Schwartz M.W., Neifing J.L., Ward W.K., Beard J.C., Palmer J.P. (1993). Quantification of the relationship between insulin sensitivity and beta-cell function in human subjects. Evidence for a hyperbolic function. Diabetes.

[24] Breda E., Cavaghan M.K., Toffolo G., Polonsky K.S., Cobelli C. (2001). Oral glucose tolerance test minimal model indexes of beta-cell function and insulin sensitivity. Diabetes.

[25] Matsuda M., DeFronzo R.A. (1999). Insulin sensitivity indices obtained from oral glucose tolerance testing: Comparison with the euglycemic insulin clamp. Diabetes Care.

[26] Abdul-Ghani M.A., Matsuda M., Balas B., DeFronzo R.A. (2007). Muscle and Liver Insulin Resistance Indexes Derived from the Oral Glucose Tolerance Test. Diabetes Care.

[27] Larsen T.M., Dalskov S., van Baak M., Jebb S., Kafatos A., Pfeiffer A., Martinez J.A., Handjieva-Darlenska T., Kunešová M., Holst C. (2010). The Diet, Obesity and Genes (Diogenes) Dietary Study in eight European countries—A comprehensive design for long-term intervention. Obes. Rev..

[28] Delgado-Lista J., Perez-Martinez P., Garcia-Rios A., Alcala-Diaz J.F., Perez-Caballero A.I., Gomez-Delgado F., Fuentes F., Quintana-Navarro G., Lopez-Segura F., Ortiz-Morales A.M. (2016). CORonary Diet Intervention with Olive oil and cardiovascular PREVention study (the CORDIOPREV study): Rationale, methods, and baseline characteristics. Am. Heart J..

[29] Wopereis S., Stroeve J.H.M., Stafleu A., Bakker G.C.M., Burggraaf J., van Erk M.J., Pellis L., Boessen R., Kardinaal A.A.F., van Ommen B. (2017). Multi-parameter comparison of a standardized mixed meal tolerance test in healthy and type 2 diabetic subjects: The PhenFlex challenge. Genes Nutr..

[30] Bates D., Mächler M., Bolker B., Walker S. (2015). Fitting Linear Mixed-Effects Models Using Lme4. J. Stat. Softw..

[31] Fox J., Weisberg S. (2019). An R Companion to Applied Regression.

[32] R Core Team (2019). R: A Language and Environment for Statistical Computing [Internet]. http://www.r-project.org/index.html.

[33] Yubero-Serrano E.M., Delgado-Lista J., Tierney A.C., Perez-Martinez P., Garcia-Rios A., Alcala-Diaz J.F., Castaño J.P., Tinahones F.J., Drevon C.A., Defoort C. (2015). Insulin resistance determines a differential response to changes in dietary fat modification on metabolic syndrome risk factors: The LIPGENE study. Am. J. Clin. Nutr..

[34] Blaak E.E. (2020). Current metabolic perspective on malnutrition in obesity: Towards more subgroup-based nutritional approaches?. Proc. Nutr. Soc..

[35] Trouwborst I., Bowser S.M., Goossens G.H., Blaak E.E. (2018). Ectopic Fat Accumulation in Distinct Insulin Resistant Phenotypes; Targets for Personalized Nutritional Interventions. Front. Nutr..

[36] Pasman W.J., Memelink R.G., de Vogel-Van den Bosch J., Begieneman M.P.V., van den Brink W.J., Weijs P.J.M., Wopereis S. (2020). Obese Older Type 2 Diabetes Mellitus Patients with Muscle Insulin Resistance Benefit from an Enriched Protein Drink during Combined Lifestyle Intervention: The PROBE Study. Nutrients.

[37] de Hoogh I.M., Oosterman J.E., Otten W., Krijger A.-M., Berbée-Zadelaar S., Pasman W.J., van Ommen B., Pijl H., Wopereis S. (2021). The Effect of a Lifestyle Intervention on Type 2 Diabetes Pathophysiology and Remission: The Stevenshof Pilot Study. Nutrients.

[38] Zhyzhneuskaya S.V., Al-Mrabeh A., Peters C., Barnes A., Aribisala B., Hollingsworth K.G., McConnachie A., Sattar N., Lean M.E.J., Taylor R. (2020). Time Course of Normalization of Functional β-Cell Capacity in the Diabetes Remission Clinical Trial After Weight Loss in Type 2 Diabetes. Diabetes Care.

[39] Wing R.R., Lang W., Wadden T.A., Safford M., Knowler W.C., Bertoni A.G., Hill J.O., Brancati F.L., Peters A., Wagenknecht L. (2011). Benefits of modest weight loss in improving cardiovascular risk factors in overweight and obese individuals with type 2 diabetes. Diabetes Care.

[40] Karter A.J., Nundy S., Parker M.M., Moffet H.H., Huang E.S. (2014). Incidence of remission in adults with type 2 diabetes: The diabetes & aging study. Diabetes Care.

[41] Dutia R., Brakoniecki K., Bunker P., Paultre F., Homel P., Carpentier A.C., McGinty J., Laferrere B. (2014). Limited Recovery of β-Cell Function after Gastric Bypass Despite Clinical Diabetes Remission. Diabetes.

[42] Nagi D., Hambling C., Taylor R. (2019). Remission of type 2 diabetes: A position statement from the Association of British Clinical Diabetologists (ABCD) and the Primary Care Diabetes Society (PCDS). Br. J. Diabetes.

[43] Yu E.A., Le N.A., Stein A.D. (2021). Measuring Postprandial Metabolic Flexibility to Assess Metabolic Health and Disease. J. Nutr..

[44] Udler M.S., Kim J., von Grotthuss M., Bonàs-Guarch S., Cole J.B., Chiou J., Boehnke M., Laakso M., Atzmon G., Glaser B. (2018). Type 2 diabetes genetic loci informed by multi-trait associations point to disease mechanisms and subtypes: A soft clustering analysis. PLOS Med..

[45] Ahlqvist E., Storm P., Käräjämäki A., Martinell M., Dorkhan M., Carlsson A., Vikman P., Prasad R.B., Aly D.M., Almgren P. (2018). Novel subgroups of adult-onset diabetes and their association with outcomes: A data-driven cluster analysis of six variables. Lancet Diabetes Endocrinol..

[46] Ahlqvist E., Prasad R.B., Groop L. (2020). Subtypes of Type 2 Diabetes Determined From Clinical Parameters. Diabetes.

[47] Hall H., Perelman D., Breschi A., Limcaoco P., Kellogg R., McLaughlin T., Snyder M. (2018). Glucotypes reveal new patterns of glucose dysregulation. PLoS Biol..

[48] Vogelzangs N., van der Kallen C.J.H., van Greevenbroek M.M.J., van der Kolk B.W., Jocken J.W.E., Goossens G.H., Schaper N.C., Henry R.M.A., Eussen S.J.P.M., Valsesia A. (2020). Metabolic profiling of tissue-specific insulin resistance in human obesity: Results from the Diogenes study and the Maastricht Study. Int. J. Obes..

[49] van der Kolk B.W., Vogelzangs N., Jocken J.W.E., Valsesia A., Hankemeier T., Astrup A., Saris W.H.M., Arts I.C.W., van Greevenbroek M.M.J., Blaak E.E. (2019). Plasma lipid profiling of tissue-specific insulin resistance in human obesity. Int. J. Obes..

[50] World Health Organization & International Diabetes Federation (2006). Definition and Diagnosis of Diabetes Mellitus and Intermediate Hyperglycaemia: Report of a WHO/IDF Consultation.

