# Supplementary material for: Effects of a 13-Week Personalized Lifestyle Intervention Based on the Diabetes Subtype for People with Newly Diagnosed Type 2 Diabetes
Source: Biomedicines. 2022 Mar 10;10(3):643. doi: 10.3390/biomedicines10030643 (PMC8945461; doi:10.3390/biomedicines10030643)
Supplement: Supplementary file 1 [file biomedicines-10-00643-s001.zip › biomedicines-1585263-supplementary.pdf]

Supplemental table S1. Deviating indices and associated treatment plan for all seven type 2 diabetes subtypes and the 'healthy' subgroup

Group	Diabetes indices	Treatment plan
HIR	Normal BCF & Liver IR	1 week VLDC and 12 wk LCD<br>Dutch Norm physical activity
MIR	Normal BCF & Muscle IR	Isocaloric diet<br>Resistance training 3x 60 min/week
CIR	Normal BCF & Combined IR	1 week VLDC and 12 wk LCD<br>Resistance training 3x 60 min/week
PB	Poor BCF & no IR	13 wk LCD<br>Dutch Norm physical activity
PB-HIR	Poor BCF & Liver IR	13 wk LCD<br>Dutch Norm physical activity
PB-MIR	Poor BCF & Muscle IR	Isocaloric diet<br>Resistance training 3x 60 min/week
PB-CIR	Poor BCF & Combined IR	13 wk LCD<br>Resistance training 3x 60 min/week
H	Healthy (normal BCF & no IR)	Excluded from study participation

HIR = moderate BCF & liver IR; CIR = moderate BCF & combined IR; PB = low BCF & no IR; PB-HIR = low BCF & liver IR; PB-CIR = low BCF & combined IR; H = healthy, i.e. no IR and moderate BCF; IR = insulin resistance; BCF = beta-cell function; VLCD = very low calorie diet; LCD = low calorie diet.

Supplemental table S2. Means (SD) and significant changes in variables after 13 weeks of intervention, and one or two years follow-up for usual care and intervention group.

Variable	Usual care	Intervention	p-value (group*time)
Bodyweight (kg)			
Baseline	90.4 (15.1)	96.3 (16.1)	
13 weeks	91.2 (15.7)	88.1 (16.9) ‡	<0.0001
1 year	90.3 (15.5)	89.3 (16.6) ‡	
2 years	87.8 (14.0) †	88.9 (13.8) ‡	
FPG (mmol/L)			
Baseline	8.3 (4.0)	7.0 (1.5)	
13 weeks	7.4 (1.6)	6.2 (0.9)	0.2595
1 year	7.6 (1.8)	6.4 (1.0)	
2 years	7.4 (1.6)	6.5 (1.4)	
HbA1c (mmol/mol)			
Baseline	49.7 (13.9)	42.6 (7.4)	
13 weeks	48.1 (9.8)	38.6 (4.9) ‡	0.0009
1 year	48.3 (10.8)	39.1 (6.7) ‡	
2 years	48.6 (10.8)	41.4 (8.9) †	

Data are mean ± standard deviation. HbA1c = glycated hemoglobin; FPG = fasting plasma glucose.
** = p<0.05, † = p<0.01 and ‡ = p<0.001 as compared to baseline.*
